# High-performance asymmetric supercapacitor made of NiMoO_4_ nanorods@Co_3_O_4_ on a cellulose-based carbon aerogel

**DOI:** 10.3762/bjnano.11.18

**Published:** 2020-01-21

**Authors:** Meixia Wang, Jing Zhang, Xibin Yi, Benxue Liu, Xinfu Zhao, Xiaochan Liu

**Affiliations:** 1 Shandong Key Laboratory for Special Silicon-containing Material, Advanced Materials Institute, Qilu University of Technology (Shandong Academy of Sciences), Jinan, 250014, P. R. China

**Keywords:** carbon aerogel, hierarchically porous structure, nanoporous material, NiMoO_4_, supercapacitor

## Abstract

In this study, a new nanoporous material comprising NiMoO_4_ nanorods and Co_3_O_4_ nanoparticles derived from ZIF-67 supported by a cellulose-based carbon aerogel (CA) has been successfully synthesized using a two-step hydrothermal method. Due to its chemical composition, the large specific surface and the hierarchical porous structure, the NiMoO_4_@Co_3_O_4_/CA ternary composite yields electrodes with an enhanced specific capacitance of 436.9 C/g at a current density of 0.5 A/g and an excellent rate capability of 70.7% capacitance retention at 5.0 A/g. Moreover, an advanced asymmetric supercapacitor (ASC) is assembled using the NiMoO_4_@Co_3_O_4_/CA ternary composite as the positive electrode and activated carbon as the negative electrode. The ASC device exhibits a large capacitance of 125.4 F/g at 0.5 A/g, a maximum energy density of 34.1 Wh/kg at a power density of 208.8 W/kg as well as a good cyclic stability (84% after 2000 cycles), indicating its wide applicability in energy storage. Finally, our results provide a general approach to the construction of CA and MOF-based composite materials with hierarchical porous structure for potential applications in supercapacitors.

## Introduction

In recent years, in the light of environmental degradation and an increasing energy demand, the development of various new, renewable and clean energy conversion and storage devices has attracted wide attention [1–4]. Among them, supercapacitors are promising candidates for energy storage owing to their advanced charge/discharge properties, the high power density and their long life cycles [5–6]. Based on the charge storage mechanism, supercapacitors can be classified into electrical double layer capacitors (EDLCs), the capacitance of which originates from the electrostatic adsorption of reversible ions at the electrode/electrolyte interface, and pseudocapacitors, for which the capacitance arises from reversible Faradaic reactions correlating with electroactive species [7–8]. Compared to EDLCs, pseudocapacitors can provide a much higher specific capacitance as a result of rapid reversible redox reactions [9–10]. Recently, advanced electrode materials based on transition metal molybdates such as NiMoO_4_ [11], CoMoO_4_ [12], MnMoO_4_ [13] and FeMoO_4_ [14] with suitable oxidation states and unique electrochemical properties are regarded as very promising materials for pseudocapacitors [15–16]. Particularly, NiMoO_4_ has been widely applied in high-performance pseudocapacitors due to its enhanced electrochemical properties resulting from the high electrochemical activity of the Ni ion and the superb electrical conductivity of the Mo ion [17–19]. Unfortunately, despite the fact that NiMoO_4_ has a high theoretical capacitance, its widespread practical application in supercapacitors is still restricted due to its low practical capacitance as well as the poor rate performance and wettability. Therefore, the construction of an integrated hierarchical porous nanoarchitecture by combining two metal oxides is a brilliant way to greatly enhance the overall electrochemical performance owing to synergistic effects [20]. For example, Li et al. synthesized 3D hybrid Co_3_O_4_/NiMoO_4_ nanowire/nanosheet arrays on a carbon cloth, which exhibited a capacitance of 3.6 F/cm^2^ at 3 mA/cm^2^, a capacitance retention of 82% and an increase of the current density from 3 to 15 mA/cm^2^ [21]. Cai et al. reported a facile two-step hydrothermal method to synthesize unique 3D Co_3_O_4_/NiMoO_4_ core/shell nanowire arrays on Ni foam, and the resulting Co_3_O_4_/NiMoO_4_ hybrid electrode exhibited an areal capacitance of 5.7 F/cm^2^ at a current density of 30 mA/cm^2^ [22]. Zhang et al. described the fabrication of 3D hierarchical Co_3_O_4_/NiMoO_4_ flower-like hybrid arrays on Ni foam with a high specific capacitance of 636.8 C/g at a current density of 5 mA/cm^2^ and a capacitance retention of 84.1% after 2000 cycles [23]. Metal-organic frameworks (MOFs) with high porosity and tunable functionality are ideal sacrificial templates to synthesize metal oxides [24–26]. As a MOF derivative, Co_3_O_4_ derived from the zeolitic imidazolate framework-67 (ZIF-67) is considered to be a good electrode material. It does not only maintain the original shape of the MOF, but also has a porous structure, which can yield a graded porous structure when combined with NiMoO_4_. Consequently, such a hierarchical porous nanoarchitecture can not only increase the specific surface area but also provide 3D pathways for fast electrolyte ion diffusion and electron transport.

To date, Ni foam [27], copper grid [28] and titanium mesh [29] have been mostly selected as collectors, whereas the high cost of these materials limited their practical application. Carbon aerogel (CA) has been considered an ideal supporting material to hybridize with electroactive materials because of its low cost, easy fabrication, large surface area, interconnected porosity and high electrical conductivity [30–31]. Due to its micro/mesoporous 3D morphology with large open pores, it offers more space to grow electroactive materials, efficiently reducing the internal resistance and enhancing the rate capability. Therefore, novel hybrid nanorods and nanoparticles of the electroactive metal oxides incorporated into a porous, conductive 3D network of CA could be promising electrode materials for supercapacitors.

Based on the above considerations, we present a simple scalable strategy to fabricate an integrated NiMoO_4_@Co_3_O_4_ hierarchical porous structure aligned on CA, which is derived from a cellulose precursor, to be applied in an advanced asymmetric supercapacitor (ASC). The NiMoO_4_ nanorods originated from ZIF-67 and were uniformly grown on CA frameworks to support the loading with Co_3_O_4_ polyhedral nanocrystals. The hierarchical porous structure of the composite made of NiMoO_4_ nanorods and Co_3_O_4_ nanoparticles derived from ZIF-67 on the CA skeleton (NiMoO_4_@Co_3_O_4_/CA) provides a reaction interface adequate for shortening of the ion diffusion length and effectively buffering the volume change in the electrochemical reaction process. More importantly, the specific surface area is enlarged for such a porous structure, facilitating reactions of active substances and improving the electrochemical performance. Our results indicate an improved energy and power density and cycle stability of the corresponding ASC device (called NiMoO_4_@Co_3_O_4_/CA//AC).

## Results and Discussion

**Figure 1:**
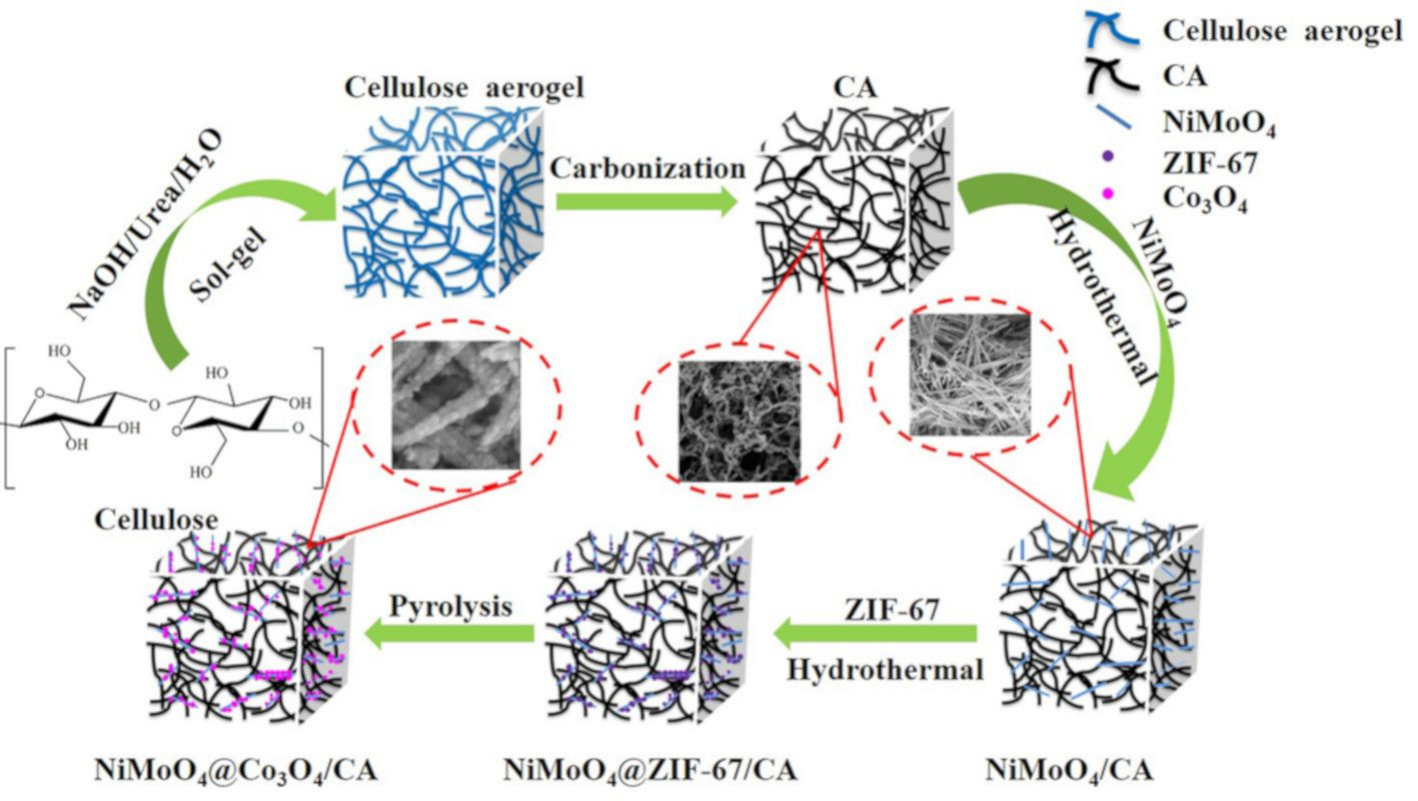
Schematic illustration of the preparation of the uniform hierarchical NiMoO_4_@Co_3_O_4_/CA sample.

The synthesis procedure of the NiMoO_4_@Co_3_O_4_/CA composite is illustrated in Figure 1. First, CA was obtained by carbonization of the cellulose aerogel precursor, which was produced from microcrystalline cellulose (MC). Second, the produced CA was used as the backbone for the growth of NiMoO_4_ nanorods employing a hydrothermal method followed by heat treatment. By this approach, NiMoO_4_/CA composites were obtained, in which the NiMoO_4_ nanorods uniformly filled the 3D network of CA, providing plenty of sites for coupling with ZIF-67. Third, ZIF-67 was in situ crystallized on the surface of the NiMoO_4_/CA skeleton by a hydrothermal method. Finally, after the pyrolysis of the NiMoO_4_@ZIF-67/CA precursor at 350 °C for 2 h under air atmosphere, the NiMoO_4_@Co_3_O_4_/CA composite was obtained.

**Figure 2:**
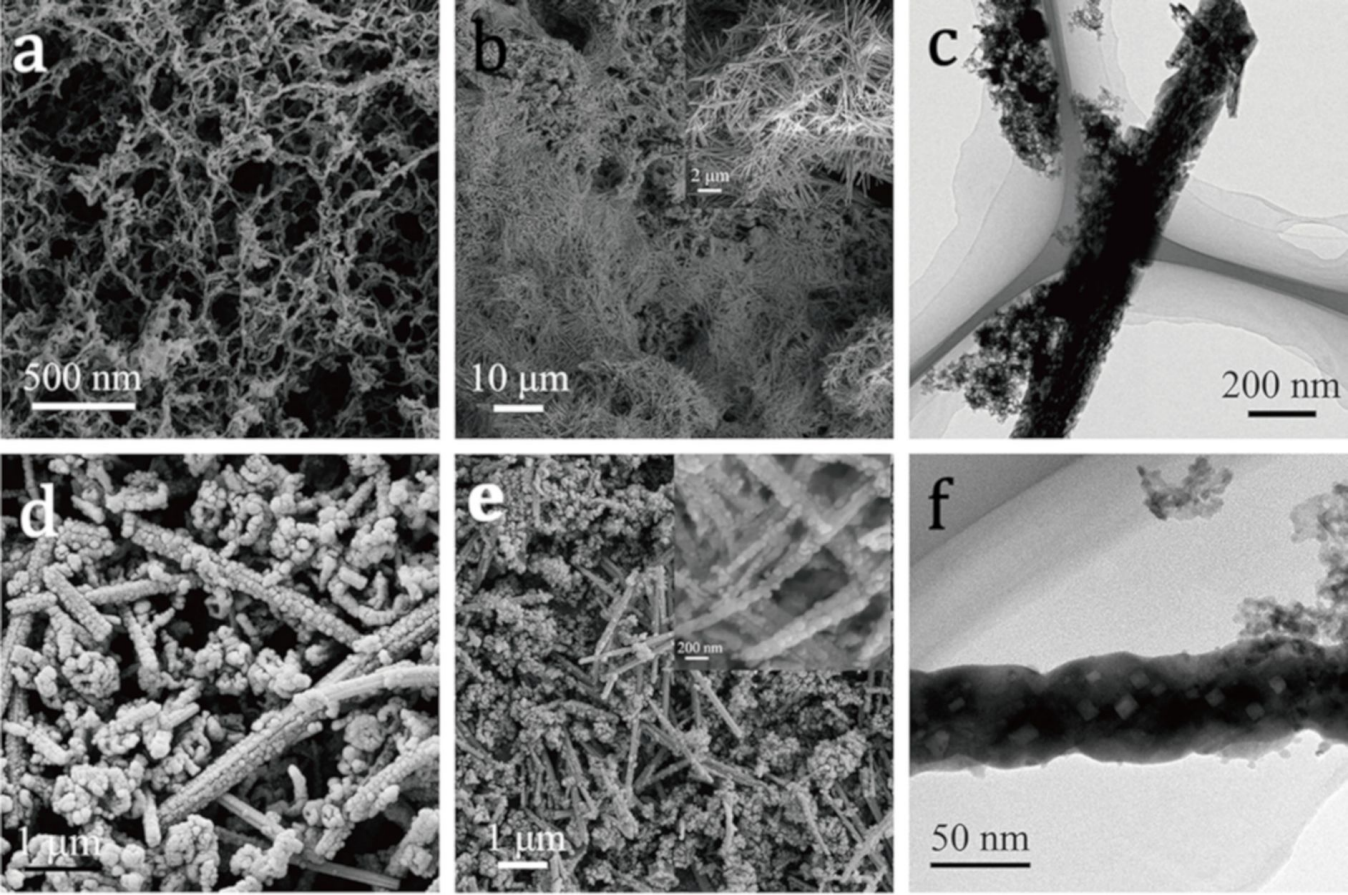
(a) SEM image of CA; (b) SEM and (c) TEM images of NiMoO_4_/CA; (d) SEM image of NiMoO_4_@ZIF-67/CA; (e) SEM and (f) TEM images of NiMoO_4_@Co_3_O_4_/CA.

The morphology of CA, NiMoO_4_/CA, NiMoO_4_@ZIF-67/CA and NiMoO_4_@Co_3_O_4_/CA was investigated by scanning electron microscopy (SEM) and transmission electron microscopy (TEM). As shown in Figure S1 in Supporting Information File 1, after the pyrolysis process, the volume of CA is drastically reduced compared to that of the precursor cellulose hydrogel and aerogel. The network of intertwined nanofibers of CA shown in Figure 2a was maintained from the cellulose aerogel (Figure S2, Supporting Information File 1). The diameter of the nanofibers was about 20–50 nm. The SEM top-view image of NiMoO_4_ is shown in Figure 2b indicating a homogenous distribution of interconnected nanofibers within the CA sample. The magnified SEM image (inset of Figure 2b) reveals that each carbon nanofiber of CA is surrounded by plenty of the NiMoO_4_ nanorods. Figure 2c confirms that the CA network is well hybridized with NiMoO_4_. This morphology supports the subsequent deposition of ZIF-67. Figure 2d and Figure 2e show that ZIF-67 with the characteristic dodecahedral morphology has been uniformly grown on the surface of the NiMoO_4_ nanorods and inside the voids of the NiMoO_4_/CA composite. ZIF-67 is processed into Co_3_O_4_ by an annealing process reported previously [32]. The resulting Co_3_O_4_ exhibits a nest-like structure and a porous morphology, and the dodecahedral structure is largely kept with no apparent collapse. The TEM image in Figure 2f clearly shows that the Co_3_O_4_ particles have preferably grown on the surface of the NiMoO_4_ nanorods with a nest-like morphology. This unique hierarchical porous architecture of the NiMoO_4_@Co_3_O_4_/CA composite is characterized by three types of network structure. The first is a filamentous network with little bundles formed by interconnected nanofibers of CA, which could provide diffusion channels for electrolyte ions and could be a conductive substrate to serve as a backbone; the second consists of interlaced NiMoO_4_ nanorods and acts as a bridge in the ternary hierarchical structure, which offers a large surface area for loading of active materials and contributes to the pseudocapacitance; the third is given by the Co_3_O_4_ nanoparticles derived from ZIF-67, which have nanosized channels and cavities.

**Figure 3:**
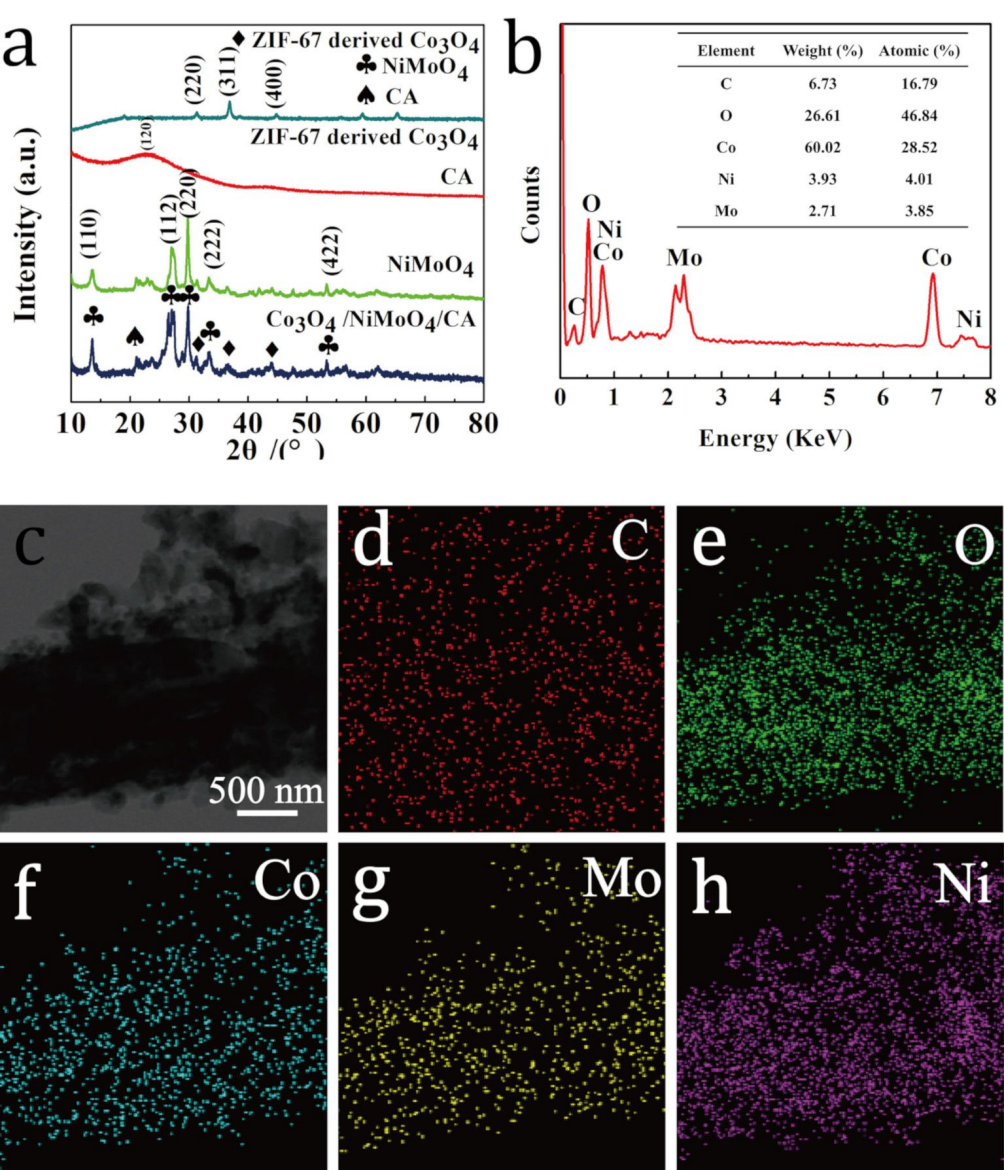
(a) XRD patterns of Co_3_O_4_, CA, NiMoO_4_ and NiMoO_4_@Co_3_O_4_/CA (from top to bottom); (b) EDS spectrum; (c) TEM image; (d–h) elemental mapping of the NiMoO_4_@Co_3_O_4_/CA sample.

The well-defined porous structure of nest-like Co_3_O_4_ provides many active sites for charge storage, thus improving the contact between electrode and electrolyte and facilitating the transport of electrons during the redox reactions [33]. Such a hierarchical structure can effectively enlarge the specific surface area of Faradaic reactions and shorten the diffusion pathways for the fast ion transfer, thus increasing the performance of the supercapacitor. The crystal structure of CA, NiMoO_4_, the Co_3_O_4_ nanoparticles derived from ZIF-67 and the NiMoO_4_@Co_3_O_4_/CA composite was examined using X-ray powder diffraction (XRD) as shown in Figure 3a. For CA, a broad diffraction peak is observed at about 22.8°, which can be attributed to the (120) planes of amorphous carbon. The five well-defined diffraction peaks appearing at 2θ values of 14.3, 25.3, 28.9, 33.7 and 53.9° are indexed to the (110), (112), (220), (222) and (422) crystal planes of NiMoO_4_, respectively, which well correspond to the standard pattern (JCPDS No. 45-0142). In addition to the characteristic reflections from CA and NiMoO_4_, the XRD pattern of Co_3_O_4_ is in good agreement with the standard pattern (JCPDS No.42-1467). The intensity of the diffraction peaks of CA and NiMoO_4_ in the XRD pattern of NiMoO_4_@Co_3_O_4_/CA is reduced due to the ZIF-67 cover on the surface of the NiMoO_4_/CA nanomaterial. To confirm the structure of NiMoO_4_@Co_3_O_4_/CA, energy-dispersive X-ray spectroscopy (EDS) and elemental mappings were carried out as shown in Figure 3b and Figure 3d–h. It can be clearly seen that there are signals for C, O, Co, Ni and Mo indicating the coexistence of the Co_3_O_4_ phase and NiMoO_4_ phase, which agrees well with the XRD results.

**Figure 4:**
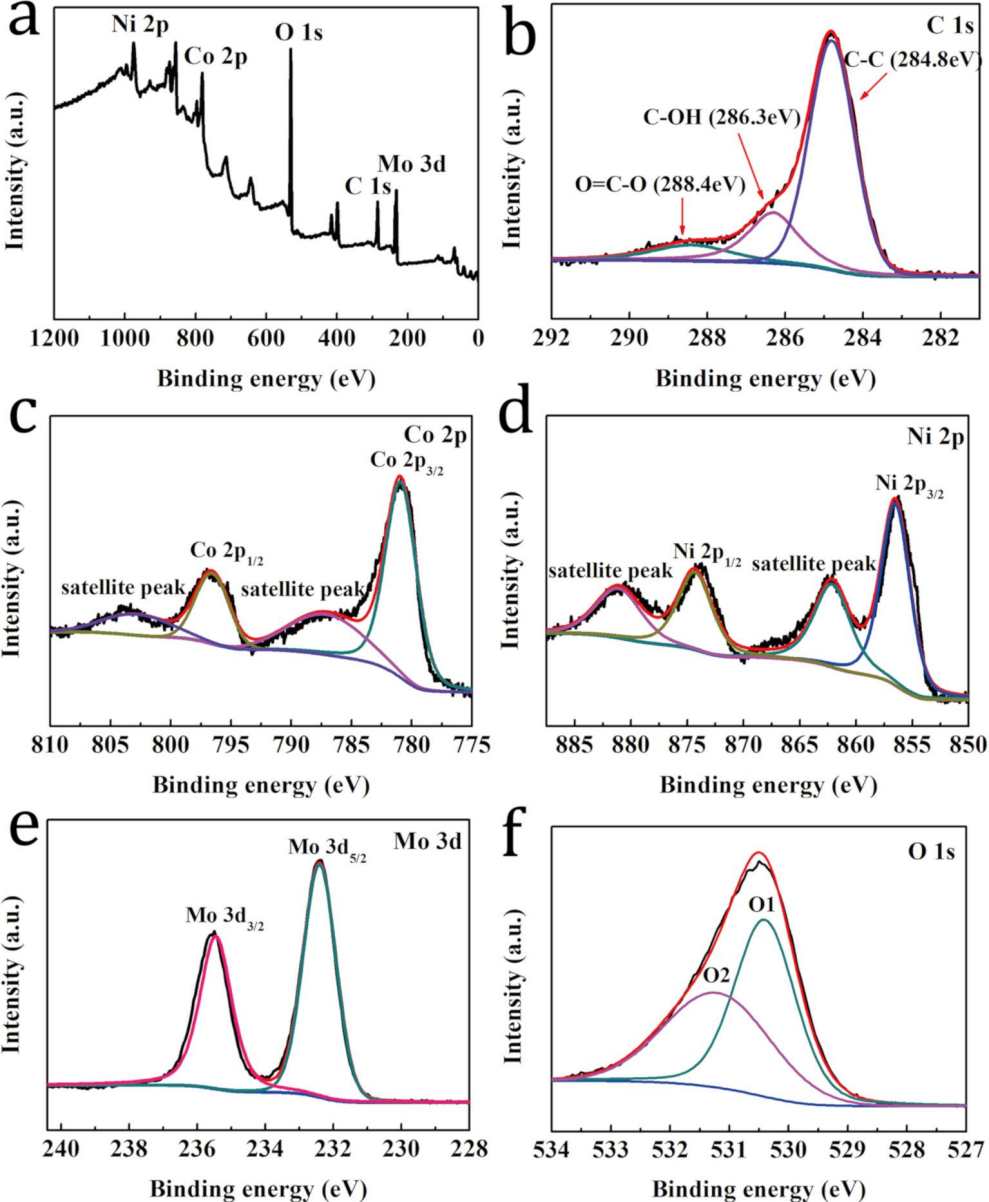
XPS spectra of the NiMoO_4_@Co_3_O_4_/CA composite: (a) Survey spectrum; (b-f) Core-level spectra of (b) C 1s, (c) Co 2p, (d) Ni 2p, (e) Mo 3d and (f) O 1s.

To investigate the chemical composition and the valence states of the NiMoO_4_@Co_3_O_4_/CA nanocomposite, X-ray photoelectron spectroscopy (XPS) was performed and the results are shown in Figure 4. According to Figure 4a, the elements Co, Ni, Mo, O and C can be clearly identified in the spectrum of the NiMoO_4_@Co_3_O_4_/CA composite. The C 1s core-level spectrum can be deconvolved into three peaks, which correspond to the C–C (284.8 eV), C–OH (286.3 eV) and O=C–O (288.4 eV) bonds (Figure 4b) [34]. In Figure 4c, two peaks are observed at 780.9 and 796.6 eV corresponding to Co 2p_3/2_ and Co 2p_1/2_, respectively, indicating that the NiMoO_4_@Co_3_O_4_/CA composite electrode material contains both Co^3+^ and Co^2+^ [35]. The peaks at 787.1 and 802.8 eV with a spin-energy separation of 15.7 eV can be attributed to the shake-up satellite peaks of Co^2+^ [36]. Figure 4d shows the Ni 2p spectrum where two characteristic peaks appear at 856.5 and 874.3 eV along with two shake-up satellite peaks with a spin-energy separation of 17.8 eV, corresponding to the Ni 2p_3/2_ and the Ni 2p_1/2_ levels of Ni^2+^ [37–38]. The Mo 3d core-level spectrum (Figure 4e) shows two main peaks at 232.4 and 235.5 eV corresponding to the Mo 3d_5/2_ and Mo 3d_3/2_ levels of Mo^6+^, respectively [39]. Figure 4f shows the core-level spectrum of O 1s. It can be divided into two main peaks at 530.4 and 531.2 eV, which are attributed to typical metal–oxygen bonds and oxygen ions of low coordination numbers at the surface, respectively [40]. The XPS results further indicate that the NiMoO_4_@Co_3_O_4_/CA sample contains C, Co, Ni, Mo and O atoms.

**Figure 5:**
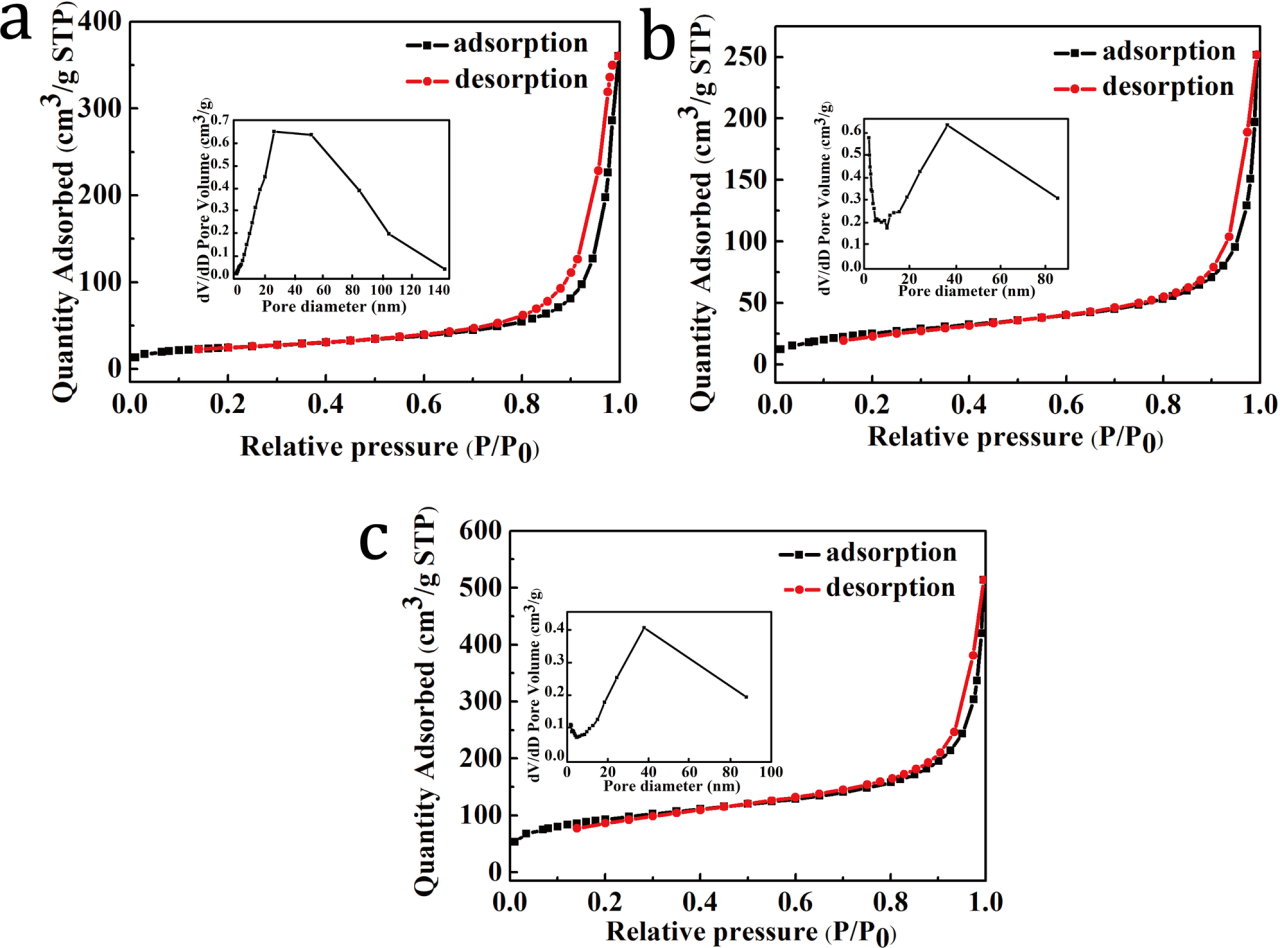
Nitrogen adsorption/desorption isotherms and pore size distribution curves of (a) CA, (b) NiMoO_4_/CA (b), and (c) NiMoO_4_@Co_3_O_4_/CA composite.

The N_2_ adsorption/desorption isotherms and the pore size distribution curves of the CA, NiMoO_4_/CA and NiMoO_4_@Co_3_O_4_/CA samples are shown in Figure 5. Both the CA and the NiMoO_4_/CA samples exhibit typical type-IV curves with distinct H_3_-type hysteresis loops suggesting the existence of mesopores. The calculated Brunauer–Emmett–Teller (BET) specific surface areas (*S*_BET_), total pore volumes and average diameters are listed in Table 1. From Figure 5c, it can be clearly seen that the NiMoO_4_@Co_3_O_4_/CA sample is characterized by a combination of type–IV and type–I isotherms, indicating the presence of micro- and mesopores with monolayer–multilayer adsorption. Furthermore, two distinct pore distribution curves are observed in the inset of Figure 5c revealing a hierarchical porosity: micro/mesopores smaller than 5 nm and meso/macropores with diameters of 20–60 nm. As shown in Table 1, the NiMoO_4_@Co_3_O_4_/CA sample has the largest *S*_BET_ of 334.47 m^2^/g and *V*_total_ of 0.8 cm^3^/g, much higher than that of CA (87.7 m^2^/g, 0.3 cm^3^/g) and NiMoO_4_/CA (94.9 m^2^/g, 0.4 cm^3^/g). The hierarchical porosity and large surface area of the NiMoO_4_@Co_3_O_4_/CA composite could be attributed to the aggregation and gathering of ZIF-67 particles on the porous skeleton of NiMoO_4_/CA forming such a porous structure, which is in agreement with the SEM images shown in Figure 2. On the one hand, the micropores mainly originate from the Co_3_O_4_ nanoparticles derived from ZIF-67. These enlarge the specific surface area and increase the number of active sites for charge storage. On the other hand, the meso/macropores consist of interconnected nanofibers of CA and interlaced NiMoO_4_ nanorods, which facilitate the inclusion of electrolytes into the particles and enhance the number of diffusion channels. Thus, we conclude that the obtained NiMoO_4_@Co_3_O_4_/CA electrode may serve as a new multi-functional material for energy conversion and storage applications [41–42].

**Table 1:** Pore structure parameters of the samples.

Sample	*S*_BET_ (m^2^/g)	*V*_total_ (cm^3^/g)	*D*_average_ (nm)

CA	87.66	0.31	24.34
NiMoO_4_/CA	94.94	0.40	14.95
NiMoO_4_@Co_3_O_4_/CA	334.47	0.82	10.81

**Figure 6:**
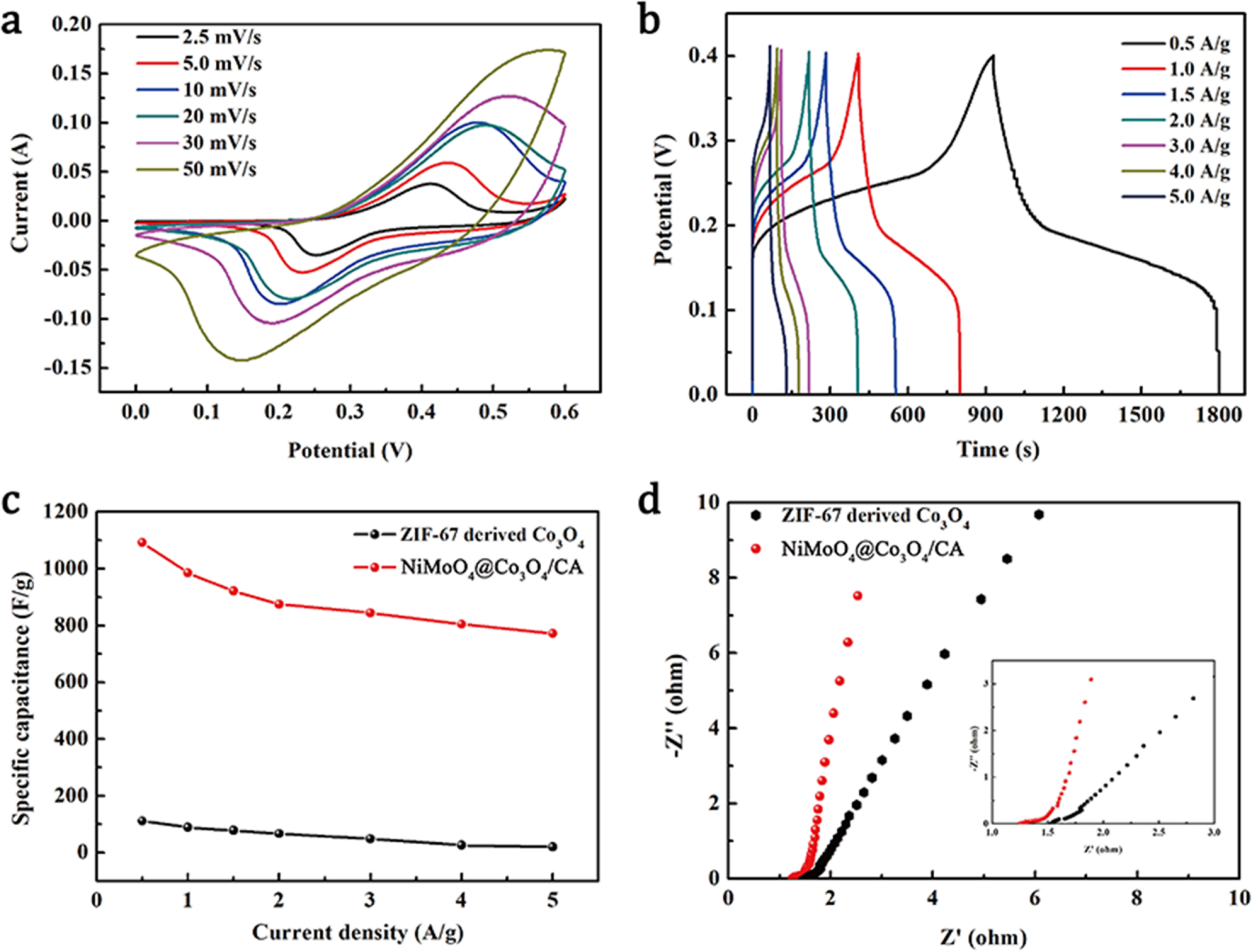
(a) Cyclic voltammetry (CV) curves at scan rates of 2.5–50 mV/s and (b) galvanostatic charge/discharge (GCD) curves at different values of the current density ranging from 0.5 to 5.0 A/g of the NiMoO_4_@Co_3_O_4_/CA ternary composite. (c) Specific capacitance at various values of the current density and (d) electrochemical impedance spectroscopy (EIS) plots of pure ZIF-67 derived Co_3_O_4_ and NiMoO_4_@Co_3_O_4_/CA electrodes.

Electrochemical measurements were performed to explore the applicability of the NiMoO_4_@Co_3_O_4_/CA electrode in a potential supercapacitor. The synthesized samples were used as working electrodes for electrochemical analysis. The electrochemical experiments were conducted by using a three-electrode testing system in a 2.0 M KOH solution. The reference electrode and the counter electrode were a saturated calomel electrode (SCE) and a platinum electrode, respectively. Figure 6a shows the cyclic voltammetry (CV) curves of the NiMoO_4_@Co_3_O_4_/CA electrode at voltage scan rates of 2.5–50 mV/s. All the CV curves shown in Figure 6a consist of a pair of strong redox current peaks, which can be attributed to the redox reaction between M^2+^ and M^3+^ (M represents Ni and Co elements) and Co^3+^ and Co^4+^, as described by the following equations [43]:

[1] $${\text{Co}}_{3}{\text{O}}_{4}+{\text{H}}_{\text{2}}\text{O}+{\text{OH}}^{-}↔3\text{CoOOH}+{\text{ e}}^{-}$$

[2] $$\text{CoOOH}+{\text{OH}}^{-}↔{\text{ CoO}}_{\text{2}}+{\text{ H}}_{\text{2}}\text{O}+{\text{ e}}^{-}$$

[3] $${\text{Ni}}^{\text{2+}}{}^{}↔{\text{ Ni}}^{\text{3+}}+{\text{ e}}^{-\;}$$

Therefore, the coexistence of Ni and Co provides multiple redox reactions for the electrochemical process. The peak currents of the CV redox peaks increased linearly with *v*^1/2^ demonstrating the battery behavior of NiMoO_4_@Co_3_O_4_/CA [44–45]. The specific capacities of NiMoO_4_@Co_3_O_4_/CA become diffusion-controlled in the high scan rate range (above 30 mV/s) and the peak current (*i*) response is proportional to the square root of the scanning rate (*i* ∼ *v*^1/2^) owing to the diffusion-controlled battery behavior. This is further confirmed by the linear change of the current of the CV redox peaks as a function of *v*^1/2^ (Figure S3, Supporting Information File 1).

To further evaluate the charge storage ability of the electrodes, galvanostatic charge/discharge (GCD) measurements were carried out within a potential window of 0–0.4 V at various values of the current density. Figure 6b shows the GCD curves of the NiMoO_4_@Co_3_O_4_/CA composite at different values of the current density. A distinct plateau occurred during each discharge process in accordance with the battery characteristics derived from the CV results. Based on the GCD curves, the specific capacitance values of the NiMoO_4_@Co_3_O_4_/CA ternary composite are calculated to be 436.9, 394.1, 368.6, 349.9, 337.8, 321.8 and 308.9 C/g at 0.5, 1.0, 2.0, 3.0, 4.0 and 5.0 A/g, respectively. These values are far larger than the capacitance values of the pure Co_3_O_4_ nanoparticles derived from ZIF-67 (44.6, 35.5, 31.3, 26.6, 19.5, 10.5 and 8.0 C/g at 0.5, 1.0, 2.0, 3.0, 4.0 and 5.0 A/g, respectively). At a high current density of 5.0 A/g, the specific capacitance of the NiMoO_4_@Co_3_O_4_/CA composite was still as high as 308.9 C/g, surpassing many metal oxides under the same conditions. For comparison, the NiMoO_4_/CA electrode was tested under the same conditions yielding a capacitance of 339.5 C/g at 0.5 A/g. Hence, the NiMoO_4_@Co_3_O_4_/CA electrode exhibited a higher specific capacitance (436.9 C/g) than the electrode of pure NiMoO_4_/CA (339.5 C/g) at a current density of 0.5 A/g (Figure S4, Supporting Information File 1). The high capacitance of the NiMoO_4_@Co_3_O_4_/CA composite may be explained as follows. Firstly, the CA scaffold acts as a conducting pathway in the NiMoO_4_@Co_3_O_4_/CA composite due to its excellent electrical conductivity and the 3D carbon nanonetwork. Secondly, NiMoO_4_ nanorods with extremely high specific capacitance are considered ideal spots for the nucleation and the growth of ZIF-67 particles. Lastly, the Co_3_O_4_ particles derived from ZIF-67 possess a nest-like structure and a porous morphology, which yields a large surface area, rich active sites and shorter diffusion paths for the Faradaic reaction.

In Figure 6d, in the low-frequency region, the NiMoO_4_@Co_3_O_4_/CA composite shows a higher phase angle than the Co_3_O_4_ particles derived from ZIF-67, indicating that the NiMoO_4_@Co_3_O_4_/CA electrodes impede the diffusion less strongly, which is attributed to the faster transport of electrons and the charge transfer resistance (*R*_ct_). Fitting the electrochemical impedance spectroscopy (EIS) plots based on the equivalent circuit model (inset of Figure 6d), reveals a solution resistance (*R*_s_) of the NiMoO_4_@Co_3_O_4_/CA composite of 1.3 Ω. This value is smaller than that of the Co_3_O_4_ particles derived from ZIF-67 with 1.5 Ω, demonstrating that the electrical conductivity is enhanced upon to the introduction of NiMoO_4_/CA. The cyclic stability of NiMoO_4_@Co_3_O_4_/CA was tested over 5000 cycles at 0.5 A/g. The capacitance retention still reaches 82.7%, while the capacitance retention of Co_3_O_4_/CA derived from ZIF-67 is only 47.1% as shown in Figure S5, Supporting Information File 1. This indicates that the NiMoO_4_@Co_3_O_4_/CA electrode has an outstanding cyclic stability, which is very important for ASC applications.

**Figure 7:**
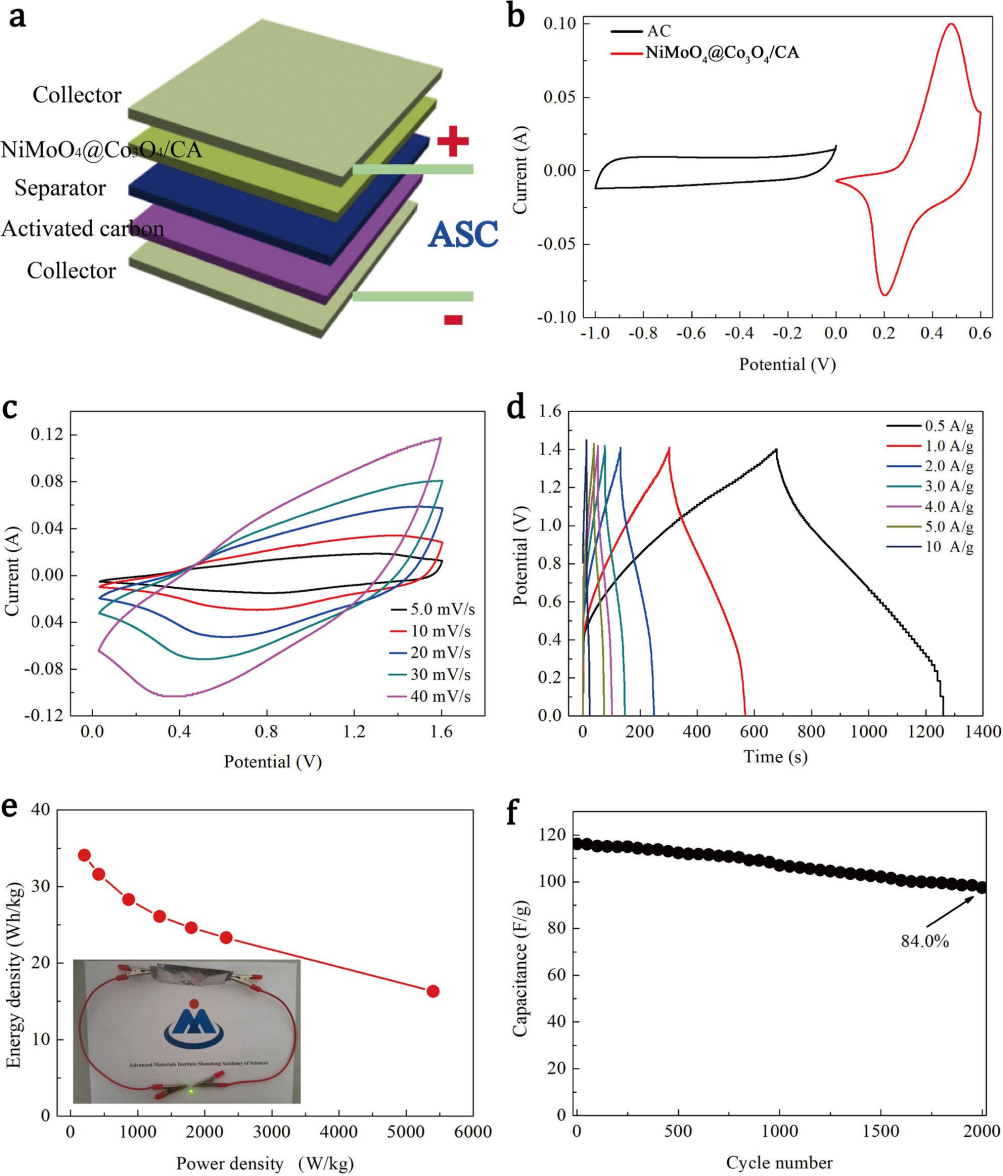
Electrochemical performance of the NiMoO_4_@Co_3_O_4_/CA//AC ASC. (a) Schematic illustration of the ASC device; (b) CV curves of the AC and NiMoO_4_@Co_3_O_4_/CA electrodes at a scan rate of 5.0 mV/s; (c) CV curves at different scan rates; (d) GCD curves at different values of the current density; (e) Ragone plot; the inset shows the green LED powered by the assembled ASC device; (f) cycle performance of the ASC device at a current density of 0.5 A/g.

A schematic diagram of the ASC device (called NiMoO_4_@Co_3_O_4_/CA//AC), which was fabricated using the NiMoO_4_@Co_3_O_4_/CA composite as the positive electrode and activated carbon (AC) as the negative electrode, is shown in Figure 7a. To determine the best working voltage window, the CV curves of the AC and the NiMoO_4_@Co_3_O_4_/CA electrodes have been measured at a scan rate of 5.0 mV/s as shown in Figure 7b. Obviously, the AC electrode works well in a voltage window between −1.0 and 0 V, while the NiMoO_4_@Co_3_O_4_/CA electrode works at 0–0.6 V with capacitive behavior. Thus, the total extent of the voltage window of the ASC device reaches 1.6 V, which is the sum of the voltage window of the AC and the NiMoO_4_@Co_3_O_4_/CA electrodes. Figure 7c exhibits the CV curves of the NiMoO_4_@Co_3_O_4_/CA//AC ASC at different scan rates from 2.5 to 50 mV/s. Obviously, the overall capacitance of the NiMoO_4_@Co_3_O_4_/CA//AC ASC device is a combination of the Faradaic pseudocapacitance and the capacitance of the EDLC [46]. The shape of the CV curve displays the characteristics of hybrid ASCs, and there is no obvious distortion with increasing scanning rate. This indicates a reliable behavior of the capacitance of the ASC device. As demonstrated in Figure 7d, the GCD curve was also evaluated to further discuss the performance of the ASC device at up to 1.4 V. Based on the GCD curves, the specific capacitance of the NiMoO_4_@Co_3_O_4_/CA//AC ASC device can reach 125.4 F/g at a current density of 0.5 A/g, and it still retains 59.8 F/g at a high current density of 10.0 A/g, indicating that the ASC cell possesses a good rate capability. The energy density and the power density of the NiMoO_4_@Co_3_O_4_/CA//AC ASC are estimated to further confirm the electrochemical properties of the device. The Ragone plot of the energy and the power density of the ASC cell is shown in Figure 7e. The energy density of the NiMoO_4_@Co_3_O_4_/CA//AC ASC increases from 16.3 to 34.1 Wh/kg as the power density decreases from 5403.3 to 208.8 W/kg. These values are significantly higher than those recently reported for ASC cells such as NiCo_2_O_4_@NiMoO_4_ NMSAs//AC [47], Co_3_O_4_@NiMoO_4_//AC [48], MNMO//AC [49], CNM-1//AC [50]. The inset in Figure 7e and Figure S6 in Supporting Information File 1 show a green LED powered by the assembled ASC device. Furthermore, the ASC device shows an outstanding cyclic performance at a current density of 0.5 A/g. As shown in Figure 7f, the capacitance retention is 84.0% of the initial value after 2000 cycles. These results demonstrate that the NiMoO_4_@Co_3_O_4_/CA//AC ASC is a very promising candidate for practical applications in high-power energy devices.

## Conclusion

A nanoporous NiMoO_4_@Co_3_O_4_/CA ternary composite has been successfully synthesized by a simple two-step hydrothermal method. The resulting NiMoO_4_@Co_3_O_4_/CA electrode shows a large specific capacitance of 436.9 C/g at 0.5 A/g, and the synergy effect of the three components is of great significance for this outstanding electrochemical performance. The ASC device based on NiMoO_4_@Co_3_O_4_/CA and AC exhibits a large capacitance of 125.4 F/g at a current density of 0.5 A/g, a maximum energy density of 34.1 Wh/kg at a power density of 208.8 W/kg, a maximum power density of 5405.3 W/kg at an energy density of 16.3 Wh/kg and an excellent cycle stability with a capacitance retention of 84.0% after 2000 cycles. The impressive performance of the ASC device is attributed to the synergistic effect of the good conduction capability of the porous 3D structure derived from CA and the large specific capacitance contributed by the transition metal oxides. Our results suggest that the NiMoO_4_@Co_3_O_4_/CA ternary composite is a quite promising pseudocapacitor material for supercapacitors.

## Experimental

### Materials

Cobalt nitrate hexahydrate (Co(NO_3_)_2_·6H_2_O), nickel nitrate hexahydrate (Ni(NO_3_)_2_·6H_2_O), sodium molybdate dihydrate (Na_2_MoO_4_·2H_2_O), 2-methylimidazole (2-MeIM) and microcrystalline cellulose (MC) (particle size: 50 μm) were purchased from Aladdin Chemical Reagent Co. Ltd. Sodium hydroxide (NaOH), urea, methanol and anhydrous ethanol were purchased from Sinopharm Chemical Reagent Co. Ltd. AC was purchased from Fujian Xinsen Carbon Industry Co. Ltd. All chemical reagents were used without further processing. In all experiments deionized water was used.

### Synthesis of MC based CA

CA was produced from MC as follows. MC was immersed in 7% NaOH/12% urea aqueous solutions precooled at −12 °C, in which the MC content was 5.0 wt %. Subsequently, the resulting mixture was kept under vigorous stirring for 20 min and then dripped into a small beaker and stored at 75 °C for 6 h to form hydrogels. Then, the produced hydrogels were kept in ethanol for 7 days, and subsequently dried under supercritical CO_2_ to obtain cellulose aerogel. Finally, CA was obtained by carbonization of the cellulose aerogel at 800 °C for 2 h under N_2_ atmosphere using a heating rate of 2 °C /min.

### Preparation of NiMoO_4_/CA

The CA described above was used as the backbone for the growth of NiMoO_4_ nanorods. Therefore, 4 mmol Ni(NO_3_)_2_·6H_2_O and 4 mmol Na_2_MoO_4_·2H_2_O were dissolved in 50 mL of deionized water to form a light-green solution. The solution was kept at room temperature for 1 h to form a homogeneous dispersion. The CA was soaked in the above solution and then transferred into a 100 mL autoclave kept at 150 °C for 6 h. Once the reaction was completed, the yellow-black products were collected by filtration and washed with deionized water for several times. Finally, the dried precipitates were annealed at 400 °C for 2 h under air to obtain the NiMoO_4_/CA samples.

### Fabrication of the NiMoO_4_@Co_3_O_4_/CA ternary composite

The in situ crystallization of ZIF-67 on the surface of NiMoO_4_/CA was carried out as follows. Typically, 1 mmol Co(NO_3_)_2_·6H_2_O and 4 mmol 2-MeIM were dissolved in 25 mL methanol. The solutions were mixed under vigorous stirring for 2 min. Then, the solutions and the activated NiMoO_4_/CA were placed in a Teflon-lined autoclave. The autoclave was kept at 100 °C for 24 h and then cooled to room temperature. The resulting ZIF-67/NiMoO_4_/CA sample was washed with anhydrous ethanol and then dried in vacuum at 80 °C for 12 h. Finally, the NiMoO_4_@Co_3_O_4_/CA samples were prepared by the pyrolysis of the NiMoO_4_@ZIF-67/CA precursors at 350 °C for 2 h under air atmosphere.

### Characterization

The crystalline structure of the prepared samples was characterized by XRD (D8 Advance, Bruker) using Cu Kα radiation (λ = 0.15406 nm) over a scan range of 5–80°. XPS (Thermo escalab 250Xi, Thermo fisher) measurements were performed using the monochromatized Al Kα radiation at 1486.6 eV. The surface morphology of the samples was observed by SEM (JSM-6701F, JEOL) at an accelerating voltage of 200 kV. TEM images and EDS mappings were recorded using a high-resolution TEM (JEOL JEM-2100) operated at an acceleration voltage of 200 kV. The pore size distribution, mean pore diameter, total pore volume and specific surface area were measured by a N_2_ adsorption analyzer (Micromeritics ASAP 2020 instrument) using the BET nitrogen adsorption/desorption technique. The pore volume was calculated from the adsorption data according to the Barrett–Joyner–Halenda (BJH) theory. The total pore volume was determined from the amount of N_2_ adsorbed at a relative pressure of *P*/*P*_0_ = 0.99.

### Electrochemical measurements

The electrochemical properties of the NiMoO_4_@Co_3_O_4_/CA electrodes were measured in a three-electrode testing system. CV, GCD and EIS were performed on the electrochemical workstation (CHI760D, Shanghai, China) in 2.0 M KOH aqueous solution. The working electrode materials were prepared by mixing the obtained sample, carbon black, acetylene black and polytetrafluoroethylene (PTEF) emulsions at a mass ratio of 80:7.5:7.5:5.0. The homogeneous slurry was coated on Ni foam substrates (1 cm × 1 cm) and dried at 80 °C for 12 h. The electrodes loaded with the hybrid were then pressed at 5.0 MPa. The SCE and platinum electrodes were used as the reference and counter electrodes, respectively. The specific capacities were calculated from the GCD curves using *C* = *I*Δ*t/m*, where *I* is the charge–discharge current, Δ*t* is the discharge time, and *m* is the mass loading of the electroactive material. The ASC device was assembled using NiMoO_4_@Co_3_O_4_/CA as the positive electrode and AC as the negative electrode. The details of preparation and characterization of the ASC device are described in the Supporting Information File 1. The cyclic stability tests were conducted on a LAND battery test system (LAND CT-2001A) at room temperature.

## Supporting Information

File 1 Details of the preparation of the ASCs, photographs and SEM images of the cellulose aerogel, electrochemical tests and the LED photograph of the ASCs device.

## References

[1] Jiang J, Li Y, Liu J, Huang X, Yuan C, Lou X W D (2012). Adv Mater (Weinheim, Ger).

[2] Winter M, Brodd R J (2004). Chem Rev.

[3] Yan J, Wang Q, Wei T, Fan Z (2014). Adv Energy Mater.

[4] Yu D, Goh K, Wang H, Wei L, Jiang W, Zhang Q, Dai L, Chen Y (2014). Nat Nanotechnol.

[5] Miller J R, Simon P (2008). Science.

[6] Kötz R, Carlen M (2000). Electrochim Acta.

[7] Yu Z, Tetard L, Zhai L, Thomas J (2015). Energy Environ Sci.

[8] Zhang L L, Zhao X S (2009). Chem Soc Rev.

[9] Lei Z, Christov N, Zhao X S (2011). Energy Environ Sci.

[10] Cao X, Shi Y, Shi W, Lu G, Huang X, Yan Q, Zhang Q, Zhang H (2011). Small.

[11] Cai D, Wang D, Liu B, Wang Y, Liu Y, Wang L, Li H, Huang H, Li Q, Wang T (2013). ACS Appl Mater Interfaces.

[12] Guo D, Zhang H, Yu X, Zhang M, Zhang P, Li Q, Wang T (2013). J Mater Chem A.

[13] Cui C, Xu J, Wang L, Guo D, Mao M, Ma J, Wang T (2016). ACS Appl Mater Interfaces.

[14] Senthilkumar B, Kalai Selvan R (2014). J Colloid Interface Sci.

[15] Peng S, Li L, Wu H B, Madhavi S, Lou X W D (2015). Adv Energy Mater.

[16] Chen T, Shi R, Zhang Y, Wang Z (2019). ChemPlusChem.

[17] Liu M-C, Kong L-B, Lu C, Ma X-J, Li X-M, Luo Y-C, Kang L (2013). J Mater Chem A.

[18] Haetge J, Djerdj I, Brezesinski T (2012). Chem Commun.

[19] Huang Y, Cui F, Zhao Y, Lian J, Bao J, Liu T, Li H (2018). Inorg Chem Front.

[20] Zhang P, Zhou J, Chen W, Zhao Y, Mu X, Zhang Z, Pan X, Xie E (2017). Chem Eng J.

[21] Li Y, Wang H, Jian J, Fan Y, Yu L, Cheng G, Zhou J, Sun M (2016). RSC Adv.

[22] Cai D, Wang D, Liu B, Wang L, Liu Y, Li H, Wang Y, Li Q, Wang T (2014). ACS Appl Mater Interfaces.

[23] Zhang Y, Yang Y, Mao L, Cheng D, Zhan Z, Xiong J (2016). Mater Lett.

[24] Wu H B, Lou X W (2017). Sci Adv.

[25] Salunkhe R R, Tang J, Kamachi Y, Nakato T, Kim J H, Yamauchi Y (2015). ACS Nano.

[26] Zheng S, Li X, Yan B, Hu Q, Xu Y, Xiao X, Xue H, Pang H (2017). Adv Energy Mater.

[27] Chen S, Zhang Z, Zeng W, Chen J, Deng L (2019). ChemElectroChem.

[28] Chen H, Zhou M, Wang T, Li F, Zhang Y X (2016). J Mater Chem A.

[29] Chen H, Yu L, Zhang J M, Liu C P (2016). Ceram Int.

[30] Zhuo H, Hu Y, Tong X, Zhong L, Peng X, Sun R (2016). Ind Crops Prod.

[31] Guilminot E, Fischer F, Chatenet M, Rigacci A, Berthon-Fabry S, Achard P, Chainet E (2007). J Power Sources.

[32] Chen X, Chen X, Zhang F, Yang Z, Huang S (2013). J Power Sources.

[33] Wang M-X, Zhang J, Fan H-L, Liu B-X, Yi X-B, Wang J-Q (2019). New J Chem.

[34] Chen L, Bai H, Huang Z, Li L (2014). Energy Environ Sci.

[35] Yang X, Sun H, Zan P, Zhao L, Lian J (2016). J Mater Chem A.

[36] Li J, Lu G, Wu G, Mao D, Guo Y, Wang Y, Guo Y (2014). Catal Sci Technol.

[37] Li Y, Jian J, Fan Y, Wang H, Yu L, Cheng G, Zhou J, Sun M (2016). RSC Adv.

[38] Yan J, Fan Z, Sun W, Ning G, Wei T, Zhang Q, Zhang R, Zhi L, Wei F (2012). Adv Funct Mater.

[39] Chi K, Zhang Z, Lv Q, Xie C, Xiao J, Xiao F, Wang S (2017). ACS Appl Mater Interfaces.

[40] Roginskaya Y E, Morozova O V, Lubnin E N, Ulitina Y E, Lopukhova G V, Trasatti S (1997). Langmuir.

[41] Liu J, Yang T, Wang D-W, Lu G Q, Zhao D, Qiao S Z (2013). Nat Commun.

[42] Xia W, Zou R, An L, Xia D, Guo S (2015). Energy Environ Sci.

[43] Zhao Y, Zhang P, Fu W, Ma X, Zhou J, Zhang X, Li J, Xie E, Pan X (2017). Appl Surf Sci.

[44] Gogotsi Y, Penner R M (2018). ACS Nano.

[45] Dubal D P, Chodankar N R, Qiao S (2019). Small.

[46] Li L, Zhang Y, Shi F, Zhang Y, Zhang J, Gu C, Wang X, Tu J (2014). ACS Appl Mater Interfaces.

[47] Zhang C, Xiao J, Lv X, Qian L, Yuan S, Wang S, Lei P (2016). J Mater Chem A.

[48] Ma X-J, Kong L-B, Zhang W-B, Liu M-C, Luo Y-C, Kang L (2014). Electrochim Acta.

[49] Li Y, Zhang S, Ma M, Mu X, Zhang Y, Du J, Hu Q, Huang B, Hua X, Liu G (2019). Chem Eng J.

[50] Yu D, Zhang Z, Teng Y, Meng Y, Wu Y, Liu X, Hua Y, Zhao X, Liu X (2019). J Power Sources.

