# Supplementary material for: High-performance asymmetric supercapacitor made of NiMoO4 nanorods@Co3O4 on a cellulose-based carbon aerogel
Source: Beilstein J Nanotechnol. 2020 Jan 21;11:240–51. doi: 10.3762/bjnano.11.18 (PMC7006496; doi:10.3762/bjnano.11.18)
Supplement: File 1 — Details of the preparation of the ASCs, photographs and SEM images of the cellulose aerogel, electrochemical tests and the LED photograph of the ASCs device. [file Beilstein_J_Nanotechnol-11-240-s001.pdf]

BEILSTEIN JOURNAL OF NANOTECHNOLOGY

Supporting Information

for

High-performance asymmetric supercapacitor made of $NiMoO_4$ nanorods@Co_3O_4 on a cellulose-based carbon aerogel

Meixia Wang, Jing Zhang, Xibin Yi, Benxue Liu, Xinfu Zhao and Xiaochan Liu

Beilstein J. Nanotechnol. **2020,** *11,* 240–251. doi:10.3762/bjnano.11.18

Details of the preparation of the ASCs, photographs and SEM images of the cellulose aerogel, electrochemical tests and the LED photograph of the ASCs device

License and Terms: This is a supporting information file under the terms of the Creative Commons Attribution License (https://creativecommons.org/licenses/by/4.0). Please note that the reuse, redistribution and reproduction in particular requires that the authors and source are credited.
The license is subject to the *Beilstein Journal of Nanotechnology* terms and conditions: (https://www.beilstein-journals.org/bjnano)

Preparation and characterization of the asymmetric supercapacitor (ASC)

The ASC device was assembled using $NiMoO_4$@Co_3O_4/CA as the positive electrode and AC as the negative. A piece of cellulose paper as the separator soaked in the electrolyte was sandwiched between the two electrodes. The electrochemical tests of the ASC device (denoted as $NiMoO_4$@Co_3O_4/CA//AC) was performed using 2.0 M KOH as alkaline electrolyte. Positive and negative charges should be balanced in an ASC as expressed by the formula $q^+ = q^-$, where q^+ and q^- represent the charges stored in the positive and in the negative electrode, respectively. The following equation was used to calculate q [1]:

$$q = mC_m \Delta V \qquad \text{(S1)}$$

When $q^+ = q^-$, the ratio between the masses of the positive electrode (m_+) and the negative electrode (m_-) were determined by the following equation [2]:

$$\frac{m_+}{m_-} = \frac{C_- \Delta V_-}{C_+ \Delta V_+} \qquad \text{(S2)}$$

The device specific capacitance, energy density (E) and power density (P) are calculated from the charge–discharge curve using the following equations [3, 4]:

$$C = \frac{I \times \Delta t}{m \Delta V} \qquad \text{(S3)}$$

$$E = \frac{C \times \Delta v}{2 \times 3.6} \qquad \text{(S4)}$$

$$P = \frac{3600 \times E}{\Delta t} \qquad \text{(S5)}$$

Where C (F/g) is the specific capacitance, I (A) is the current, Δt (s) is the discharge time, ΔV (V) is the working voltage window, m (g) is the mass of the active material, E (Wh/kg) is the energy density and P (W/kg) is power density.

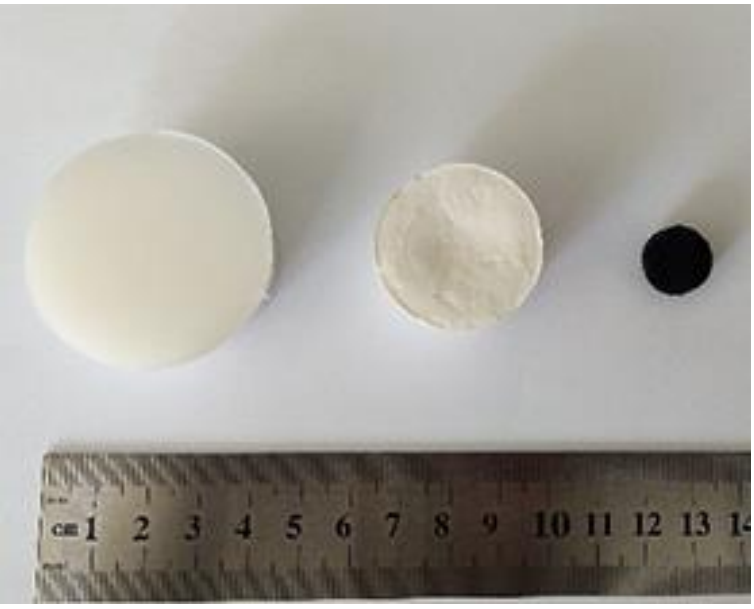

Figure S1: Digital photograph of the cellulose hydrogel, the cellulose aerogel and CA.

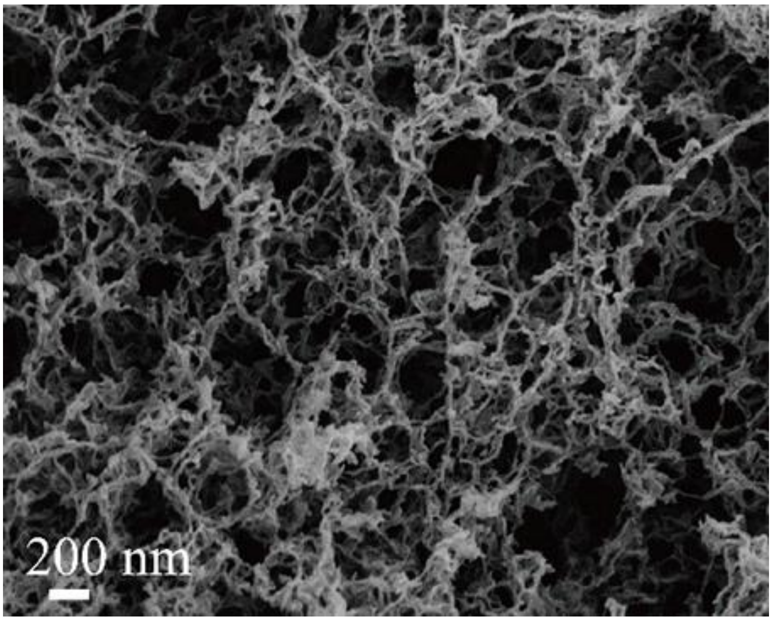

Figure S2: SEM of the cellulose aerogel.

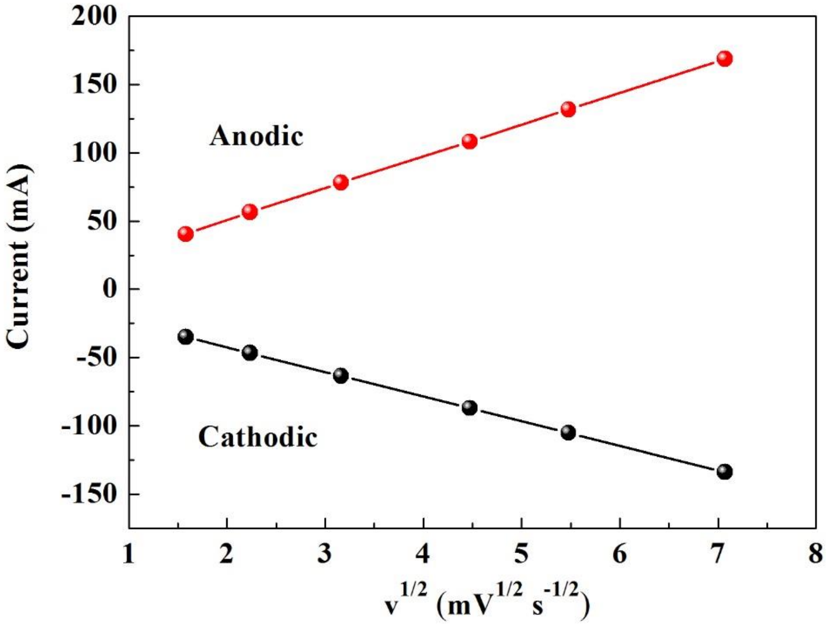

Figure S3: Linear fit of the experimental values of the current of the CV redox peaks as a function of $v^{1/2}$ of the $NiMoO_4@Co_3O_4$/CA composite.

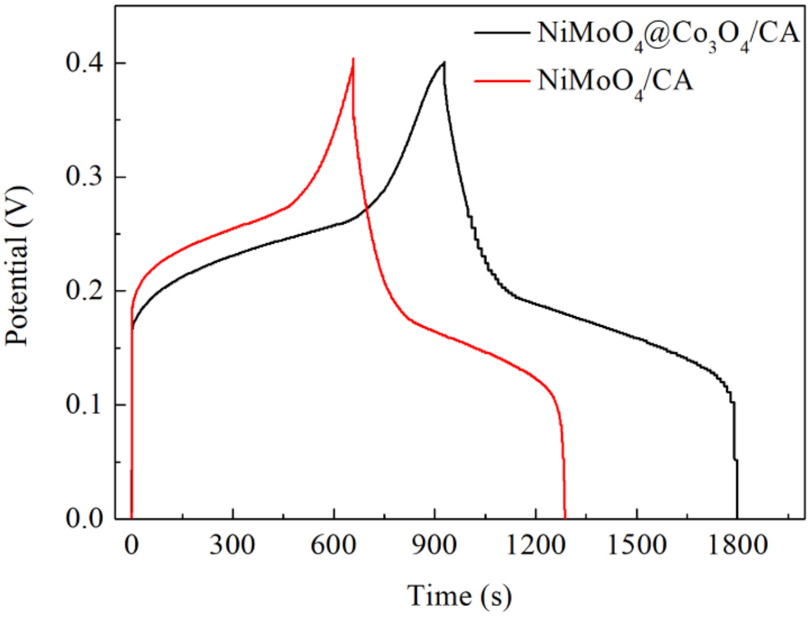

Figure S4: GCD curves of the $NiMoO_4$/CA and the $NiMoO_4@Co_3O_4$/CA at a current density of 0.5 A/g.

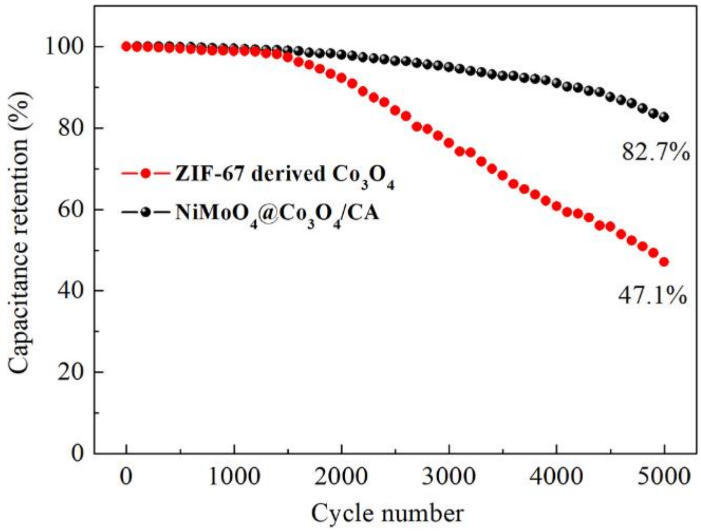

Figure S5: Cycle performance of ZIF-67 derived Co_3O_4/CA and $NiMoO_4$@Co_3O_4/CA at a current density of 0.5 A/g.

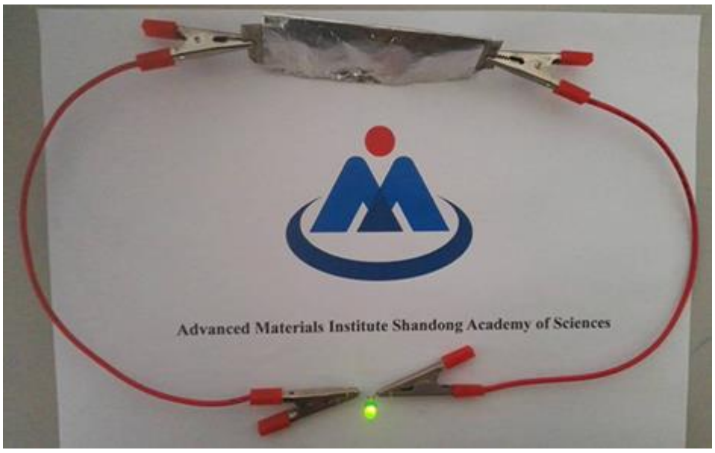

Figure S6: LED lighting photograph of the ASC device

References

1. Chen S.; Chen H.; Li C.; Fan M.; Lv C.; Tian G.; Shu K. *J. Mater. Sci*., **2017**, *52*, 6687-6696. doi:10.1007/s10853-017-0903-2

2. Salunkhe R. R.; Lin J.; Malgras V.; Dou S. X.; Kim J. H.; Yamauchi Y. *Nano Energy*, **2015**, *11*, 211-218. doi:10.1016/j.nanoen.2014.09.030

3. Liu M. C.; Xu Y.; Hu Y. X.; Yang Q. Q.; Kong L. B.; Liu W. W.; Niu W. J.; Chueh Y. L. *ACS Appl. Mater. Interfaces*, **2018**, *10*, 35571-35579. doi:10.1021/acsami.8b09085

4. Zhang J. T.; Jiang J. W.; Li H. L.; Zhao X. S. *Energy Environ. Sci.*, **2011**, *4*, 4009-4015. doi: 10.1039/C1EE01354H
